# Supplementary material for: Acetazolamide as an Add-on Therapy Following Barbed Reposition Pharyngoplasty in Obstructive Sleep Apnea: A Randomized Controlled Trial
Source: Life (Basel). 2024 Jul 31;14(8):963. doi: 10.3390/life14080963 (PMC11355526; doi:10.3390/life14080963)
Supplement: Supplementary file 1 [file life-14-00963-s001.zip › life-3062480-supplementary.pdf]

life MDPI

Supplemental Files

Supplemental methods

Study design and participant flow

26 patients were randomized to receive either BRP + acetazolamide or BRP + placebo. Baseline characteristics of both groups after randomization are summarized in Table S1. There were no significant differences between both groups at baseline. Of the 26 patients who participated in the study, twenty-one completed the entire trial (9 with acetazolamide and 12 with placebo). Three patients initially allocated to the acetazolamide group chose not to initiate medication anymore and subsequently withdrew their participation. Following the recovery period post BRP, these individuals no longer had an interest in pursuing additional pharmacotherapy. Additionally, a fourth patient who was assigned to the acetazolamide group later decided to discontinue their participation in the study due to the excessive burden of medication-related side effects. In the placebo group, one participant similarly elected to terminate involvement in the study for analogous reasons. Participant flow is summarized in figure 1 in the main paper.

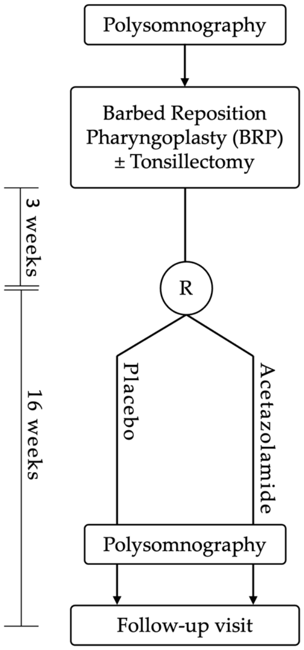

Figure S1. Study design flowchart. R: randomization.

Table S1. Baseline characteristics of both treatment groups after randomization.

	BRP + Acetazolamide (n = 13)	BRP + Placebo (n = 13)	p-value
	Demographics and anatomical features		
Age, years	44.0 (34.5-59.5)	51 (41-61)	0.281
Gender, male (%)	12 (92)	11 (85)	0.999
BMI, kg/m^2	28.5 (25.7-30.5)	28.5 (27.1-29.9)	0.758
	**Comorbidities**		
Smoking, No. (%)	5 (38.4)	3 (23.1)	0.673
Hypertension, No. (%)	2 (15.4)	3 (23.1)	1.000
Heart failure, No. (%)	0 (0)	0 (0)	1.000
Asthma, No. (%)	0 (0)	1 (7.7)	1.000

COPD, No. (%)	0 (0)	0 (0)	1.000
Diabetes mellitus, No. (%)	0 (0)	0 (0)	1.000
Baseline polysomnographic parameters			
TST, min	424 (377-447)	427 (369-454)	0.898
SEI, %	86.0 (77.7-90.5)	86.6 (73.7-93.0)	0.701
Mean SpO_2, %	93.8 (92.5-95.1)	94.0 (93.5-95.1)	0.644
T90%, %TST	2.3 (0.4-15.0)	2.3 (0.3-5.3)	0.663
ODI_3, events/h	22.1 (15.3-40.3)	17.4 (13.9-25.5)	0.399
AHI, events/h	25.2 (20.6-46.7)	22.4 (19.6-34.1)	0.259
SASHB, (%min/h)	43.2 (26.4-63.1)	43.1 (23.8-63.8)	0.931
Patient reported symptom scores			
ESS	8.0 (5.5-14.5)	11.0 (6.0-14.5)	0.537
FOSQ-10	15.9 (13.3-19.2)	14.7 (11.1-18.0)	0.281
VAS snoring	8.5 (6.3-9.8)	8 (6-9.8)	0.679

Groups were compared using a Mann-Whitney U test and Fisher's Exact test as appropriate. Abbreviations: BMI = body mass index; TST = total sleep time; SEI = sleep efficiency index; SpO2 = oxygen saturation; T90 = sleep time with oxygen saturation ≤ 90%; ODI = oxygen desaturation index; AHI = apnea-hypopnea index; SASHB = sleep apnea-specific hypoxic burden; ESS = Epworth sleepiness scale; FOSQ-10 = functional outcome of sleep questionnaire; VAS = visual analogue scale.

Supplemental results and discussion

Analyses with outlier included

Outlier analysis was performed on the change in AHI. This analysis revealed a singular outlier within the placebo group (age: 45 years, BMI: 30.7 kg/m^2, delta AHI: 388%). Subsequent investigations into this patient's data failed to identify the cause for this significant increase. The main paper describes analyses with the outlier included. For the sake of completeness, analyses were also carried out without the outlier (Table S2).

Table S2. Baseline and follow-up polysomnographic results and questionnaire scores of both treatment groups with outlier excluded.

	BRP + Acetazolamide (*n* = 9)			BRP + Placebo (*n* = 11)		
	**Baseline**	**Follow-up**	***p***	**Baseline**	**Follow-up**	***p***
Sleep characteristics						
TST, min	424 (356-446)	414 (408-454)	0.426	392 (366-447)	427 (415-456)	0.054
SEI, %	82.8 (73.5-89.6)	84.7 (81.7-90.5)	0.426	81.3 (72.6-91.8)	87.7 (84.8-89.6)	0.052
REM, %TST	20.2 (14.7-23.3)	24.5 (18.0-30.2)	0.059	20.0 (16.7-21.6)	21.0 (17.4-27.5)	0.054
N1, %TST	11.5 (5.0-16.0)	5.2 (3.0-9.5)	0.020	12.6 (2.2-20.9)	6.3 (3.8-12.7)	0.181
N2, %TST	61.8 (46.6-65.1)	51.6 (49.0-60.9)	0.129	54.3 (45.5-59.6)	55.2 (50.2-59.7)	0.269
N3, %TST	11.7 (6.8-21.3)	17.2 (12.1-26.0)	0.164	15.8 (0.0-21.0)	16.5 (8.0-21.1)	0.311
Mean SpO_2, % (*)	94.1 (92.4-95.5)	95.2 (93.8-95.7)	0.008	94.0 (93.3-95.1)	94.1 (93.2-94.4)	0.537
Nadir SpO_2, %	86 (83.5-89.0)	89.0 (87.3-90.8)	0.219	85 (83-89)	86.1 (84.0-88.0)	0.219
T90%, %TST (*)	2.3 (0.3-7.8)	0.2 (0.0-3.3)	0.016	2.3 (0.1-3.9)	1.5 (0.7-2.1)	0.186
ODI_3, events/h (*)	22.1 (12.2-39.0)	9.0 (5.6-15.0)	0.004	21.3 (12.5-26.8)	14.9 (9.0-26.0)	0.264
AHI, events/h (*)	25.2 (20.6-47.8)	7.7 (5.3-14.5)	0.004	22.8 (19.8-35.5)	13.8 (9.9-31.2)	0.014
AHIsupine, events/h	51.6 (36.9-67.3)	27.5 (9.1-53.2)	0.074	51.4 (38.6-73.1)	29.2 (16.7-54.0)	0.083
AHInonsupine, events/h	19.1 (2.3-29.0)	4.1 (2.0-8.8)	0.055	9.4 (0.0-18.2)	7.3 (3.3-25.7)	0.981
OAHI, events/h (*)	23.9 (20.5-46.6)	7.6 (5.0-13.8)	0.004	21.2 (19.4-35.3)	13.5 (6.8-27.7)	0.014
CAHI, events/h	0.2 (0.0-1.3)	0.3 (0.0-0.8)	0.219	0.0 (0.0-0.8)	0.1 (0.0-0.8)	0.984
SASHB, (%min/u) (*)	36.3 (26.4-62.2)	10.3 (7.9-18.0)	0.008	43.1 (25.1-63.2)	27.7 (15.6-47.1)	0.032
Patient reported symptom scores						
ESS	8.0 (4.0-15.5)	5.0 (2.0-10.5)	0.164	11.0 (7.0-15.0)	6.5 (2.3-10.5)	0.063

FOSQ-10	18.0 (14.1-19.5)	19.4 (18.1-20.0)	0.102	14.7 (11.6-18.3)	17.7 (14.8-19.0)	0.025
VAS snoring (*)	8.0 (6.3-9.8)	3.0 (1.5-4.0)	0.008	7.5 (6.0-10)	4.5 (3.0-9.0)	0.117

The p-values for before-after comparisons were determined using the Wilcoxon signed-rank test. A Mann-Whitney U test was conducted to compare the pre-post treatment differences between the placebo and acetazolamide group for each outcome. The presence of a statistically significant difference between the two groups, indicating that the acetazolamide group significantly differs from the placebo group, is denoted by an asterisk (*). Abbreviations: TST = total sleep time; SEI = sleep efficiency index; REM = rapid eye movement; N = non-REM sleep stages 1-3; SpO2 = oxygen saturation; T90 = sleep time with oxygen saturation ≤ 90%; ODI = oxygen desaturation index; AHI = apnea-hypopnea index; OAHI = obstructive apnea-hypopnea index; CAHI = central apnea-hypopnea index; SASHB = sleep apnea-specific hypoxic burden; ESS = Epworth sleepiness scale; FOSQ-10 = functional outcome of sleep questionnaire; VAS = visual analogue scale.

OSA endotypes

Calculations of OSA-specific endotypes before and after treatment were also conducted for experimental analysis. As already mentioned in the discussion, contrary to expectations, no reduction in loop gain was demonstrated in the acetazolamide group. Also in the placebo group, loop gain did not change following surgery (Table 3, Figure S2). This, however, aligns with previous research where surgical interventions did not result in a decrease in loop gain [1,2]. One study, however, did demonstrate a reduction in loop gain after surgery for OSA [3]. A plausible explanation for these contradictory finding is that these patients had a more severe form of sleep apnea with a high baseline loop gain. Severe OSA, generally coupled with significant hypoxemia, can induce chemoreflex changes that elevate loop gain [4,5]. As a result, adequate treatment of severe OSA may also lead to a decrease in loop gain, as also shown using CPAP therapy [6].

Although not statistically significant within each group, there was a general trend towards an increase in Vpassive (p = 0.067, figure S2). This corresponds to a decrease in collapsibility after treatment. This aligns with the findings of Wong et al., who also demonstrated a reduction in collapsibility following surgery [1].

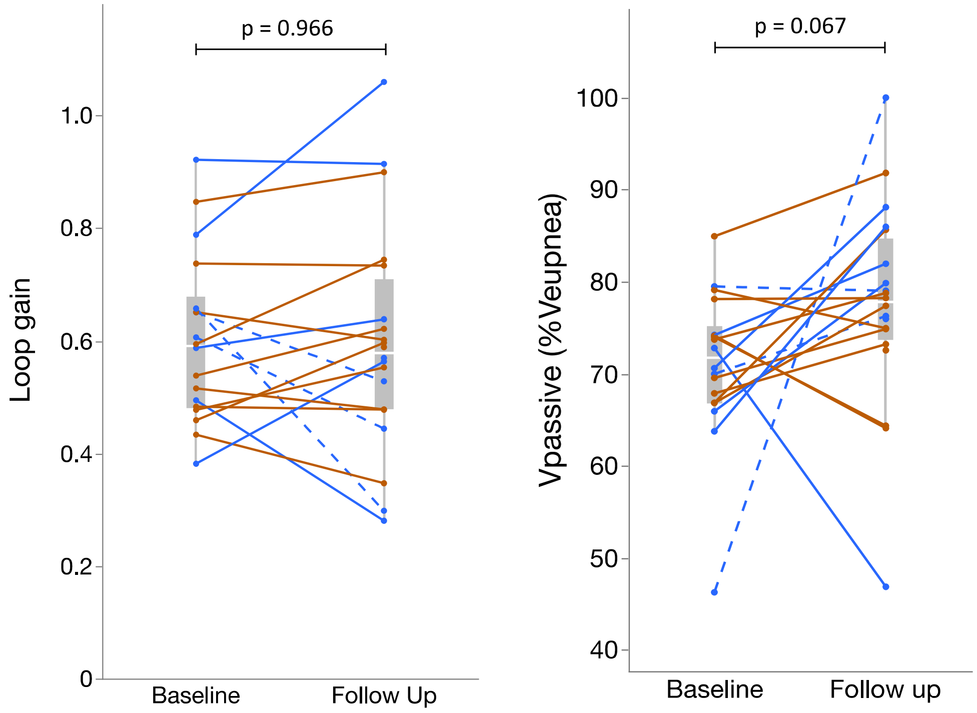

Figure S2. Loop gain (left) and Vpassive (right) before and after treatment. The orange lines indicate the placebo, while the blue lines indicate acetazolamide. Dotted lines represent patients whose

medication dosage was reduced to a single dose per day. Overall, loop gain didn't change after treatment. There is a trend towards an increase in Vpassive, however without reaching statistical significance. Calculations of endotypes were not possible for two patients (one in each treatment arm) due to the unavailability of raw PSG data.

References

1. Wong, A. M.; Landry, S. A.; Joosten, S. A.; Thomson, L. D. J.; Turton, A.; Stonehouse, J.; Mansfield, D. R.; Burgess, G.; Hays, A.; Sands, S. A.; Andara, C.; Beatty, C. J.; Hamilton, G. S.; Edwards, B. A., Examining the impact of multilevel upper airway surgery on the obstructive sleep apnoea endotypes and their utility in predicting surgical outcomes. *Respirology* **2022,** *27* (10), 890-899. [10.1111/resp.14280]
2. Joosten, S. A.; Leong, P.; Landry, S. A.; Sands, S. A.; Terrill, P. I.; Mann, D.; Turton, A.; Rangaswamy, J.; Andara, C.; Burgess, G.; Mansfield, D.; Hamilton, G. S.; Edwards, B. A., Loop Gain Predicts the Response to Upper Airway Surgery in Patients With Obstructive Sleep Apnea. *Sleep* **2017,** *40* (7), [10.1093/sleep/zsx094]
3. Li, Y.; Ye, J.; Han, D.; Zhao, D.; Cao, X.; Orr, J.; Jen, R.; Deacon-Diaz, N.; Sands, S. A.; Owens, R.; Malhotra, A., The Effect of Upper Airway Surgery on Loop Gain in Obstructive Sleep Apnea. *J Clin Sleep Med* **2019,** *15* (6), 907-913. [10.5664/jcsm.7848]
4. Edwards, B. A.; Sands, S. A.; Owens, R. L.; White, D. P.; Genta, P. R.; Butler, J. P.; Malhotra, A.; Wellman, A., Effects of hyperoxia and hypoxia on the physiological traits responsible for obstructive sleep apnoea. *J Physiol* **2014,** *592* (20), 4523-35. [10.1113/jphysiol.2014.277210]
5. Younes, M.; Ostrowski, M.; Thompson, W.; Leslie, C.; Shewchuk, W., Chemical control stability in patients with obstructive sleep apnea. *Am J Respir Crit Care Med* **2001,** *163* (5), 1181-90. [10.1164/ajrccm.163.5.2007013]
6. Salloum, A.; Rowley, J. A.; Mateika, J. H.; Chowdhuri, S.; Omran, Q.; Badr, M. S., Increased propensity for central apnea in patients with obstructive sleep apnea: effect of nasal continuous positive airway pressure. *Am J Respir Crit Care Med* **2010,** *181* (2), 189-93. [10.1164/rccm.200810-1658OC]
